# Prognosis and personalized medicine prediction by integrated whole exome and transcriptome sequencing of hepatocellular carcinoma

**DOI:** 10.3389/fgene.2023.1075347

**Published:** 2023-02-02

**Authors:** Debao Li, Lei Lei, Jinsong Wang, Bo Tang, Jiuling Wang, Rui Dong, Wenjiong Shi, Guo Liu, Tingting Zhao, Yuzhang Wu, Yi Zhang

**Affiliations:** ^1^ Department of Immunology, Medical College of Qingdao University, Qingdao, Shandong, China; ^2^ Chongqing International Institute for Immunology, Chongqing, China; ^3^ Institute of Immunology, PLA, Army Medical University, Chongqing, China; ^4^ Department of Ophthalmology, Airforce Hospital, Chengdu, Sichuan, China; ^5^ Qionglai Hospital of Traditional Chinese Medicine, Qionglai, Sichuan, China; ^6^ School of Pharmacy and Bioengineering, Chongqing University of Technology, Chongqing, China

**Keywords:** hepatocellular carcinoma, whole exome sequencing, RNA sequencing, TP53, prognostic model

## Abstract

Hepatocellular carcinoma (HCC) is a clinically and genetically heterogeneous disease. To better describe the clinical value of the main driver gene mutations of HCC, we analyzed the whole exome sequencing data of 125 patients, and combined with the mutation data in the public database, 14 main mutant genes were identified. And we explored the correlation between the main mutation genes and clinical features. Consistent with the results of previous data, we found that *TP53* and *LRP1B* mutations were related to the prognosis of our patients by WES data analysis. And we further explored the associated characteristics of *TP53* and *LRP1B* mutations. However, it is of great clinical significance to tailor a unique prediction method and treatment plan for HCC patients according to the mutation of *TP53*. For *TP53* wild-type HCC patients, we proposed a prognostic risk model based on 11 genes for better predictive value. According to the median risk score of the model, HCC patients with wild-type *TP53* were divided into high-risk and low-risk groups. We found significant transcriptome changes in the enrichment of metabolic-related pathways and immunological characteristics between the two groups, suggesting the predictability of HCC immunotherapy by using this model. Through the CMap database, we found that AM580 had potential therapeutic significance for high-risk *TP53* wild-type HCC patients.

## 1 Introduction

Hepatocellular carcinoma (HCC) is one of the most common malignancies, accounting for 85% of liver cancer. It is also the third leading cause of cancer-related death; morbidity and mortality are increasing yearly (Hoshida et al., 2009; El-Serag, 2011). Although progress has been made in the diagnosis and treatment of HCC, the high heterogeneity of HCC still makes prognosis prediction and treatment challenges, and the 5-year overall survival rate of HCC patients is still less than 20% (Long et al., 2017; Nault and Villanueva, 2021). Therefore, there is an urgent need for molecular characterization of HCC to help the discovery of biomarkers, to promote proper diagnosis, accurate treatment, and prognosis prediction, then to achieve the purpose of precision medicine, tiered care, and accurate prediction in a real sense. The continuous innovation of sequencing technology provides us with many HCC data resources. We can better understand HCC by integrating data from different sources, which is helpful in finding clinical therapeutic targets and biomarkers (Ding et al., 2019). To date, genome analysis in HCC has revealed a wide diversity of genetic changes, including DNA, transcribed mRNA, and non-coding RNA (Craig et al., 2017; Sun and Malhotra, 2018). In this genomic era, the emergence of a large number of genome sequencing techniques and data is conducive to further cancer exploration. It is significant for early diagnosis, treatment, and prognosis monitoring of cancer (Hong et al., 2020).

Although cancer cells show similar characteristic behavior, there are almost no same mutations in individual tumors (Hanahan and Weinberg, 2011). The most frequently altered gene in human tumors is *TP53*, and mutated *TP53* is associated with poor clinical outcomes (Kandoth et al., 2013). *TP53* mutations lead to the loss of function of the wild type p53 and deprive cells of innate tumor inhibitory response; *TP53* mutations can promote tumor cell survival and adapt to various internal and external stress conditions, including overproliferation-related DNA damage, oxidative and protein toxic stress, nutritional fluctuations, physical constraints, matrix cues, and anti-tumor immune responses (Senft and Ze’ev, 2016; Mantovani et al., 2019). In HCC, *TP53* mutation is associated with clinical features such as tumor differentiation, vascular invasion, serum alpha-fetoprotein (AFP) level, and tumor stage, and *TP53* mutation is also correlated to the tumor microenvironment and immune-related characteristics of hepatocellular carcinoma (Dong et al., 2017; Biton et al., 2018; Hong et al., 2020). To sum up, it is essential to make unique prediction methods and treatments for *TP53* according to the mutation state of HCC. Currently, the development of HCC prognostic gene signature is mainly based on the whole population and patients with *TP53* mutants. There is no corresponding research to put forward the prognostic model for patients with *TP53* wild-type HCC.

In this study, combined with available clinical information, we describe the mutation map and molecular mechanism of the main driver genes in HCC through gene exon sequencing and mutation data from public databases; in addition, we tailored a prognosis prediction model for *TP53* wild-type HCC patients and screened out potential therapeutic drugs for high-risk patients.

## 2 Materials and methods

### 2.1 Patients and tumor materials

Our study included one hundred and twenty-five patients with hepatocellular carcinoma (HCC). Tumor puncture samples were collected from Qionglai Hospital of Traditional Chinese Medicine, and the whole exome sequencing was performed. The selected patients’ baseline demographic, clinical, and pathological data were collected and recorded during diagnosis, including sex, age, AFP, PIVKA-II, tumor size and number, tumor stage, treatment mode, survival status, and the latest follow-up information. The review date from the date of diagnosis to the date of death or the date of the last follow-up was collected for OS calculation, and the date from the first treatment to the time of the first recurrence was collected for PFS calculation. Exclusion criteria: when exploring whether patients with stage TNMI and TNMII relapse within one year, filter out patients with a follow-up period of less than one year; in the survival analysis of patients with stage TNMIII and TNMIV, filter out patients with an overall survival period of less than one month. Details were shown in Supplementary Table S1. The study was approved by the local ethics committee [No. 2021 (01)], and the patient’s informed consent was obtained following Chinese law.

### 2.2 Common data set

Somatic mutation data were derived from TCGA (364 HCC samples) and ICGC (904 HCC samples, including LICA-CN, LICA-FR, and LIRI-JP) databases. Gene expression data (raw counts) were derived from TCGA (375 HCC samples, 50 normal tissue samples) and ICGC (LIRI-JP, 227 hepatocellular carcinoma samples). The original counts of gene expression data were normalized to CPM, and HCC samples with gene expression data, somatic mutation data, and complete clinical information were screened for subsequent analysis. Exclusion criteria: At the time of survival analysis, patients with overall survival of less than one month and a follow-up period of fewer than two months were excluded.

### 2.3 Whole exome sequencing and data analysis

Whole exome sequencing was performed using the Agilent SureSelect Human All Exon v6 Kit (Agilent Technologies, Santa Clara, CA, United States), then sequenced on the Illumina HiSeq 2000 System (Illumina, San Diego, CA, United States) by Shanghai Personal Biotechnology Cp. Ltd. The 150bp paired reads were aligned to the human reference genome (GRCh38) by BWA (version 0.7.15). GATK (version 4.0) detected single nucleotide variants and indels. Copy number variation (CNV) was identified from the bam file by VarSeq (version 2.2.4, Golden Helix^®^). All the variants were annotated by VarSeq with the public (gnomAD, 1,000 Genomes, DGV, ICGC, TCGA) and in-house databases. Variants passed quality control (Read depth ≥6, Genotype Qualities ≥20) were filtered. Variant filtering criteria: 1. Read depth filtering. A variant will be retained only if it meets the following conditions. (1) The total reads depth ≥10; (2) The alternative reads depth ≥5; (3) The alternative reads depth/total reads depth ≥0.1.2. Variant annotation result filtering. If a site meets the following conditions, it will be filtered. (1) The variants located upstream and downstream of the gene were filtered out; (2) The variants located in the intron region were filtered out; (3) Synonymous variants were filtered out; (4) The variants located on the transcript ablation region were filtered out; (5) The variant pathogenicity classified as “LOW” or “MODIFIER” were filtered out.

### 2.4 Principal analysis tools and R packages

SPSS (Statistical Product Service Solutions, version 26.0) is used to analyze the correlation between molecular and clinical features and the statistics of clinical information. Rstudio (4.1.0) for data processing, analysis, and graphic visualization. GSEA is used for pathway enrichment analysis to determine the enrichment differences in biologically significant pathways among HCC patients in different groups. FDR <0.25, *p* < 0.05, and | NES | > 1 indicates that the results are meaningful. R package enrichplot for Gene Ontology (GO) and Jingdu Encyclopedia of Gene and Genome (KEGG) Analysis. R package Maftools is used to read and visualize MAF files, and R package GenVisR is used to draw a gene mutation waterfall map. R package edgeR and limma are used to identify differentially expressed genes (DEGs) among different groups of HCC patients. The cpm function in edgeR can convert the original data of gene expression into CPM format. R package survival and survminer are used for Kaplan-Meier survival analysis and graphical visualization. Evaluation of the predictive ability of R package timeROC and ggDCA in prognostic models. R package regplot for the Construction of Nomotu. R package ggplot2, pheatmap and ggpubr are mainly used for graphic visualization, including heat maps, histograms, volcanoes, *etc.* CIBERSORT algorithm is used to evaluate the relative proportion of 22 immune cells. The multi-algorithmic immune cell infiltration result file of the TCGA database is obtained at the website (http://timer.comp-genomics.org). The CMap database (http://clue.io/query) is used to screen small molecule drugs.

### 2.5 Statistical analysis

The correlation analysis between classified variables were processed by the chi-square or Fisher exact test. All statistical analysis was carried out using R software (version 4.1.0) or SPSS (version 26.0).

## 3 Results

### 3.1 Mutations of the main driver genes

**FIGURE 1:**
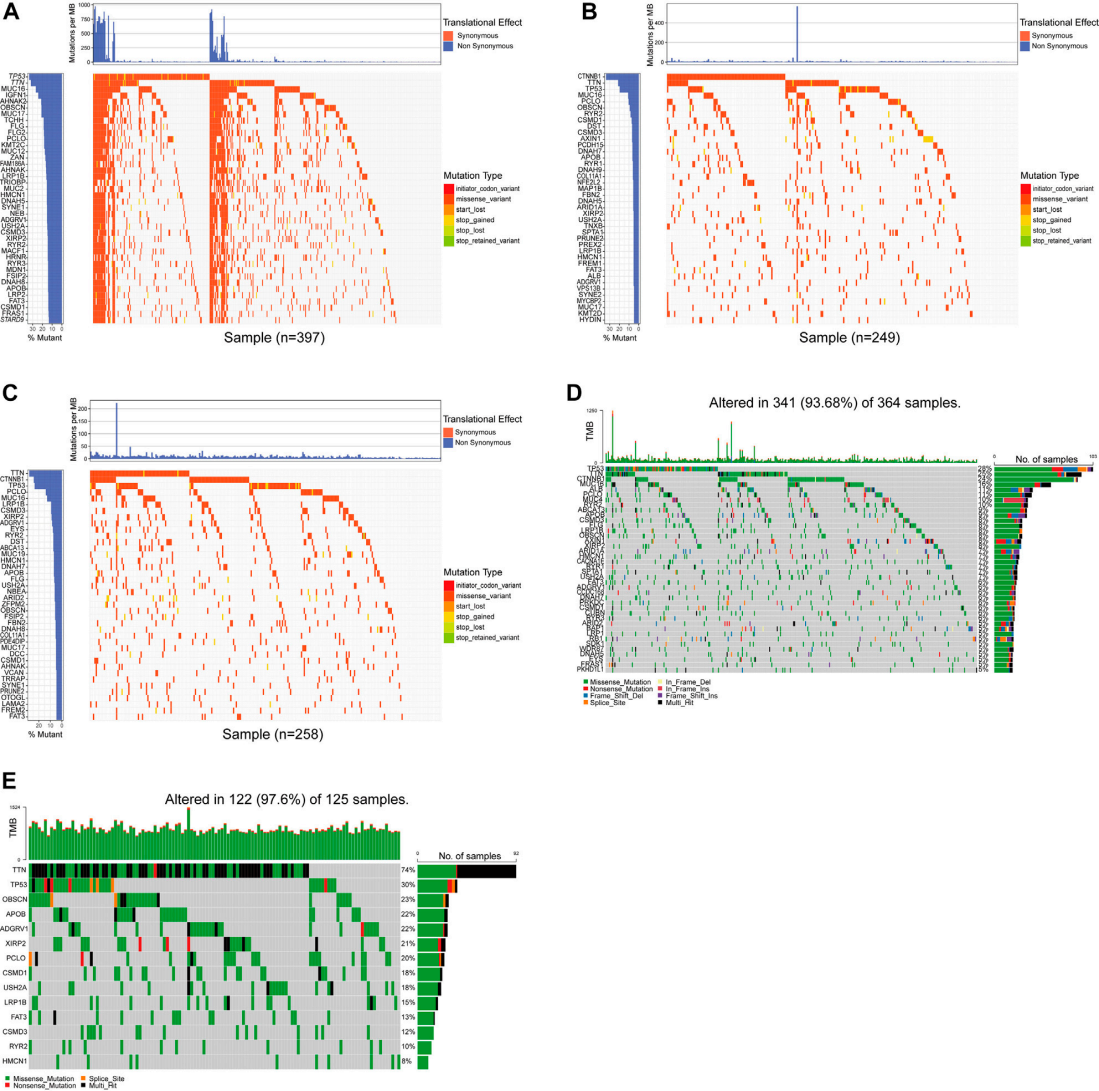
The primary mutations were screened by whole exon sequencing and a database. **(A–D)** The waterfall chart showed the top 40 genes in the four cohorts of ICGC-LICA-CN, ICGC-LICA-FR, ICGC-LIRI-JP, and TCGC-LIHC. **(E)** The mutation profiles of 14 repetitive genes in the whole exon sequencing queue.

**FIGURE 2:**
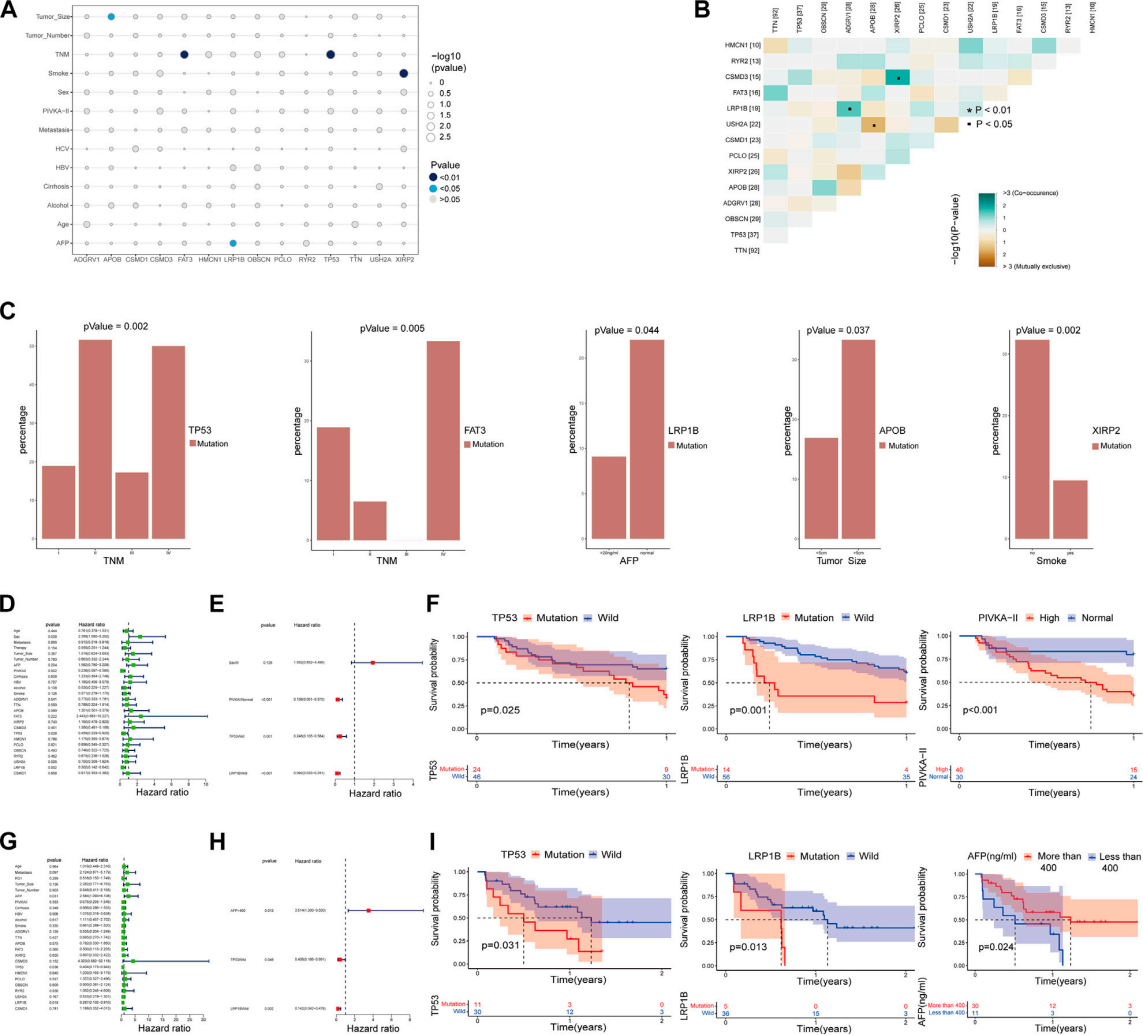
Correlation analysis of significant mutant genes with major clinical features and prognosis. **(A–C)** Correlation analysis of major mutant genes with major clinical features. **(B)** Coexistence and mutual exclusion among major mutant genes. **(D,E)** Univariate and multivariate COX regression analysis of the main influencing factors and progression-free survival (PFS) within one year in HCC patients with TNMI and TNMII. **(F)** The independent influencing factors of 1-year progression-free survival (PFS) in HCC patients with TNMI and TNMII were analyzed by K-M survival curve. **(G,H)** Univariate and multivariate COX regression analysis of the main influencing factors and overall survival (OS) in HCC patients with TNMIII and TNMIV. **(I)** The independent influencing factors of overall survival (OS) in HCC patients with TNMIII and TNMIV were analyzed by Kaplan-Meier survival curve.

The median age of 125 patients with hepatocellular carcinoma was 53 years (range: 28–78 years), and the proportion of males was high (84.8% vs. 15.2%); Tumor staging using TNM classification was TNM I in 42.4% (*n* = 53), TNM II in 24.8% (*n* = 31), TNM III in 23.2% (*n* = 29), and TNM IV in 9.6% of the cases (*n* = 12); 75 resectable patients (60%) and 50 unresectable patients (40%) received curative surgery, immunotherapy, radiofrequency ablation (RFA), palliative treatment or combined treatment (Supplementary Table S1). The mean depth of target sequencing of the samples was 105X. The percentage of the target regions with a mean depth over 20X was 99.3%. Since we only get the WES data of tumor puncture samples from HCC patients, there may be errors in directly describing the gene mutations in our cohort. To better describe the mutations of the main driver genes of hepatocellular carcinoma (HCC), we screened the top 40 genes with mutation frequency from the somatic mutation data in the ICGC database and the TCGA database, including the four cohorts of ICGC-LICA-CN, ICGC-LICA-FR, ICGC-LIRI-JP and TCGC-LIHC, respectively, among which *TTN*, *TP53*, *OBSCN*, *APOB*, *ADGRV1*, *XIRP2*, *PCLO*, *CSMD1*, *USH2A*, *LRP1B*, *FAT3*, *CSMD3*, *RYR2*, and *HMCN1* are repeated in four HCC queues (Figures 1A–D). In our cohort (N = 125), the frequencies of mutations in these genes were equally high, predominantly with missense mutations, and *TTN* was the gene with the highest mutation frequency; other genes with higher mutation frequencies were as follows: *TP53* (30%), *OBSCN* (23%), *APOB* (22%), *ADGRV1* (22%), *etc.* The mutation frequency was altered compared to the somatic mutation data for HCC in the TCGA and ICGC databases (Figure 1E). Analysis of tumor TNM stage showed that *TP53* mutations and *FAT3* mutations were distributed differently in different TNM stages (*p* < 0.05), and both were more common in patients with advanced HCC. Serum AFP level is a commonly used and vital index in diagnosing HCC and monitoring therapeutic efficacy. *LRP1B* mutations were mainly enriched in the normal serum AFP level (*p* = 0.044). We also found that *APOB* mutations were primarily distributed in HCC patients with tumors larger than 5 cm (*p* = 0.037) and *XIRP2* mutations were primarily distributed in non-smoking patients (*p* = 0.002) (Figures 2A, C). By Fisher’s exact test, we found an evident coexistence between *ADGRV1* mutations and *LRP1B* mutations, and between *XIRP2* mutations and *CSMD3* mutations (*p* < 0.05), there was significant mutual exclusion between the *APOB* mutation and the *USH2A* mutation (*p* < 0.05) (Figure 2B). In addition, we statistically analyzed mutations in main driver genes with other major clinical features and found no statistical significance (Supplementary Table S2).

### 3.2 Impact of main driver gene mutations on the prognosis of HCC patients

**FIGURE 3:**
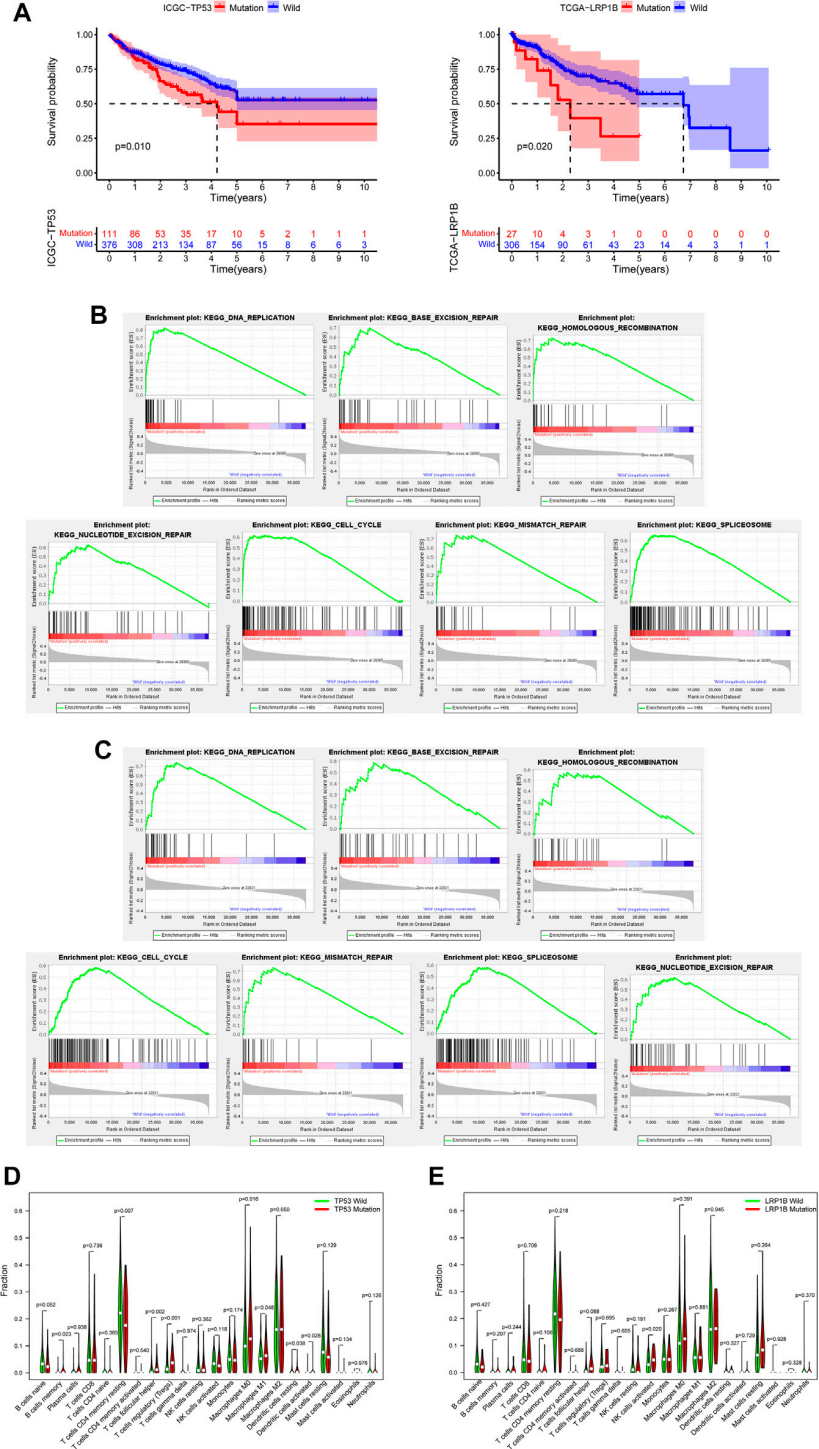
Verification of the database and biological characteristics related to TP53 mutation and LRP1B mutation. **(A)** Kaplan-Meier survival analysis was performed on the mutation data with clinical information in TCGA and ICGC databases. **(B)** GSEA enrichment analysis showed that the pathway was significantly related to *TP53* mutation. **(C)** GSEA enrichment analysis showed that the pathway was significantly related to *LRP1B* mutation. **(D)** The difference of immunocyte infiltration abundance between *TP53* mutation group and wild group in HCC patients. **(E)** The difference of immunocyte infiltration abundance between *LRP1B* mutation group and wild group in HCC patients.

To better distinguish our clinical patient samples, we focused on 1-year progression-free survival (PFS) of TNMI and TNMII HCC patients (Supplementary Table S3) and overall survival (OS) of TNMIII and TNMIV HCC patients (Supplementary Table S4). Through univariate COX regression analysis, we found that *TP53* mutations, *LRP1B* mutations, sex, and serum abnormal prothrombin II (PIVKA-II) levels were closely related to recurrence within one year in TNMI and TNMII patients (*p* < 0.05) (Figure 2D). In contrast, *TP53* mutations, *LRP1B* mutations, and serum AFP levels were closely related to death in TNMIII and IV patients (*p* < 0.05) (Figure 2G). In multivariate COX regression analysis, we found that *TP53* mutations, *LRP1B* mutations, and serum abnormal prothrombin II (PIVKA-II) levels were independent factors of recurrence in HCC patients with TNMI and TNMII within one year (Figure 2E). *TP53* mutations, *LRP1B* mutations, and serum alpha-fetoprotein (AFP) levels were independent influencing death factors in TNMIII and TNMIV HCC patients (Figure 2H). At the same time, the Kaplan-Meier survival analysis also validates our findings (Figures 2F, I). To further verify our conclusion, we performed a Kaplan-Meier survival analysis on the mutation data with complete clinical information in TCGA and ICGC database and also found that *LRP1B* mutations and *TP53* mutations were significantly correlated with the prognosis of HCC patients (Figure 3A).

### 3.3 Biological characteristics related to *TP53* mutation and *LRP1B* mutation

To further explore the biological characteristics related to *TP53* mutation and *LRP1B* mutation, we downloaded RNA sequencing data from the TCGA database and screened out samples with RNA sequencing data and somatic mutation data. According to the mutation state of *TP53* and *LRP1B*, the RNA sequencing data were divided into mutant group and wild group for follow-up analysis. Because many pathways are involved in tumorigenesis, the poor prognosis of HCC patients with *TP53* or *LRP1B* mutations may be related to the abnormal regulation of many signal pathways in HCC. Through GSEA enrichment analysis, we found seven KEGG signal pathways, including DNA replication, base excision repair, homologous recombination, cell cycle, mismatch repair, splicing, and nucleotide excision repair, showed significant differential enrichment in *TP53* or *LRP1B* mutant groups (Figures 3B, C). We then analyzed the infiltration of 22 kinds of immune cells in the tumor immune microenvironment of HCC patients. We found that memory B-cell, follicular helper T-cell, Treg cells, M0 macrophages, M1 macrophages, resident DC cells, and activated DC cells were significantly enriched in the *TP53* mutation group, and CD4 resident memory T-cell were significantly improved in the *TP53* non-mutation group (Figure 3D). Perhaps due to the small proportion of patients with *LRP1B* mutation in TCGA, we only found that activated NK cells were significantly enriched in the *LRP1B* mutation group, and the infiltration of other immune cells did not show a significant statistical difference (Figure 3E).

### 3.4 TCGA as a training set to construct a prognostic model of *TP53* wild-type HCC

**FIGURE 4:**
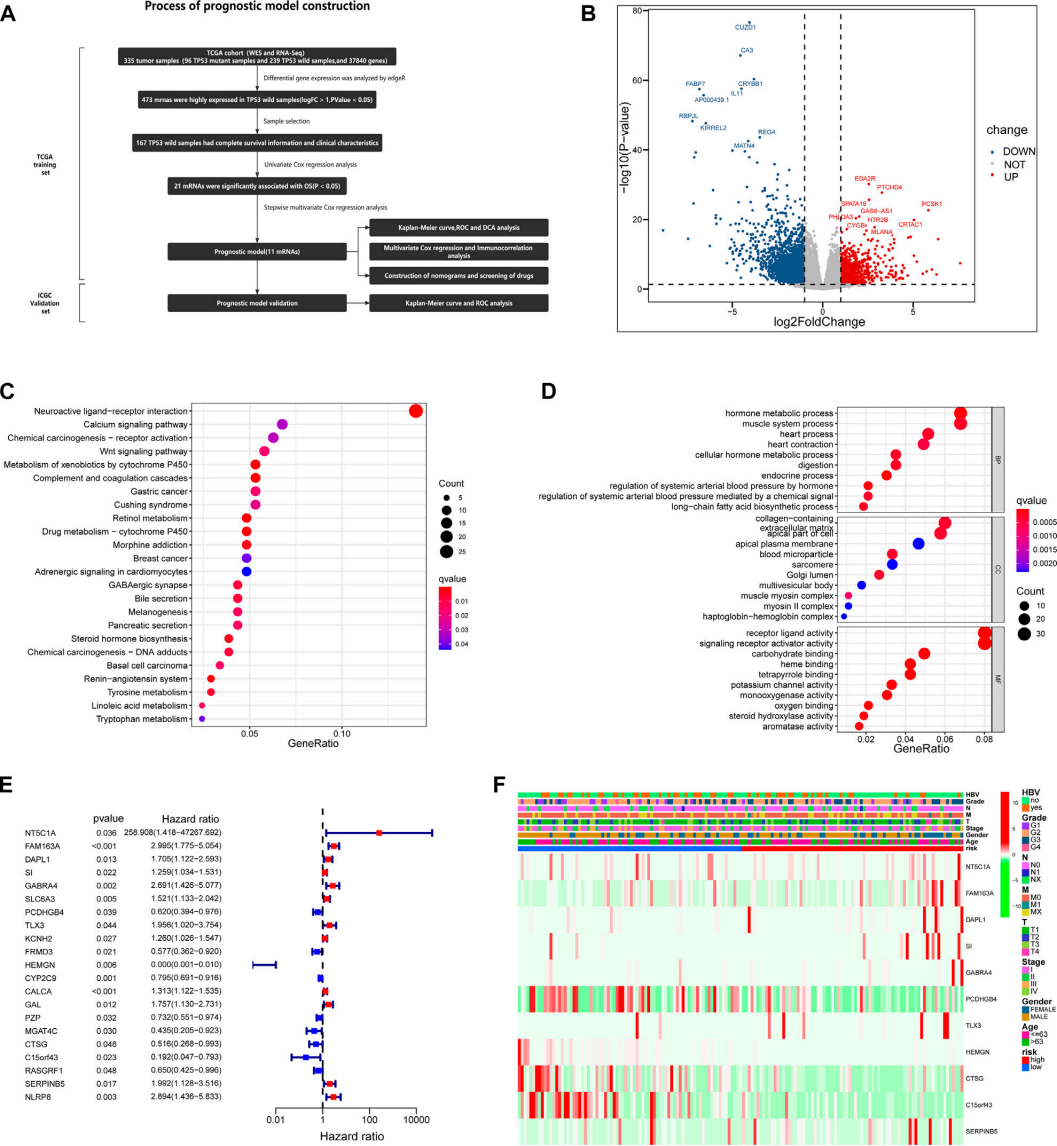
Based on TCGA database, the prognostic risk model of HCC patients with TP53 wild type was constructed. **(A)** A complete process for the development and verification of prognostic risk models. **(B)** The volcano map is used to show the differentially expressed genes between *TP53* mutant group and wild group. **(C)** Results of KEGG enrichment of highly expressed genes in *TP53* wild group. **(D)** Results of GO enrichment of highly expressed genes in *TP53* wild group. **(E)** Univariate Cox regression analysis of the relationship between highly expressed genes and OS in *TP53* wild group. **(F)** The differential expression of genes involved in the construction of prognostic risk model in different clinical feature groups and different risk groups.

We created a detailed analysis flow chart (Figure 4A) to describe our analysis process more intuitively. In the TCGA database, 335 HCC patients have both RNA sequencing and somatic mutation data. Patients with HCC were divided into groups according to *TP53* mutation status, including 96 *TP53* mutant samples and 239 *TP53* wild-type samples. Through differential gene expression analysis, we found that 809 genes were highly expressed in *TP53* wild-type samples (logFC >1, *p* < 0.05), including 473 mRNA (Figure 4B). GO enrichment analysis showed that highly expressed genes were significantly enriched in the metabolic hormone process, muscle system process, collagen-containing, extracellular matrix apical part of cell, receptor ligand activity and signaling receptor activator activity (*p* < 0.05) (Figure 4C). KEGG enrichment analysis showed that highly expressed genes were significantly enriched in Neuroactive ligand-receptor interaction and Wnt signaling pathway pathways (Figure 4D). To ensure the integrity and rationality of clinical information, 167 samples of *TP53* wild-type were included in the training set for constructing prognostic models. Through batch univariate COX regression analysis, it was found that 21 mRNA were closely related to the total survival time (OS) of patients with *TP53* wild-type HCC (Figure 4E). Then, stepwise multivariate COX analysis, 11 mRNA were selected to construct the prognosis model of *TP53* wild-type HCC patients to narrow the scope of gene screening further. The COX regression coefficient of related genes in each model is calculated based on COX multiple regression model, and the model risk score of each sample is defined as the product of the regression coefficient and the expression value of related genes in each model. According to the median risk score, the training set samples are divided into high-risk and low-risk groups for follow-up analysis. In addition, we further analyzed the distribution of the expression levels of 11 genes involved in constructing the model in different clinical characteristics, including hepatitis B virus infection, grade, stage, age, and sex (Figure 4F).

### 3.5 Evaluation and verification of prediction ability of the model

**FIGURE 5:**
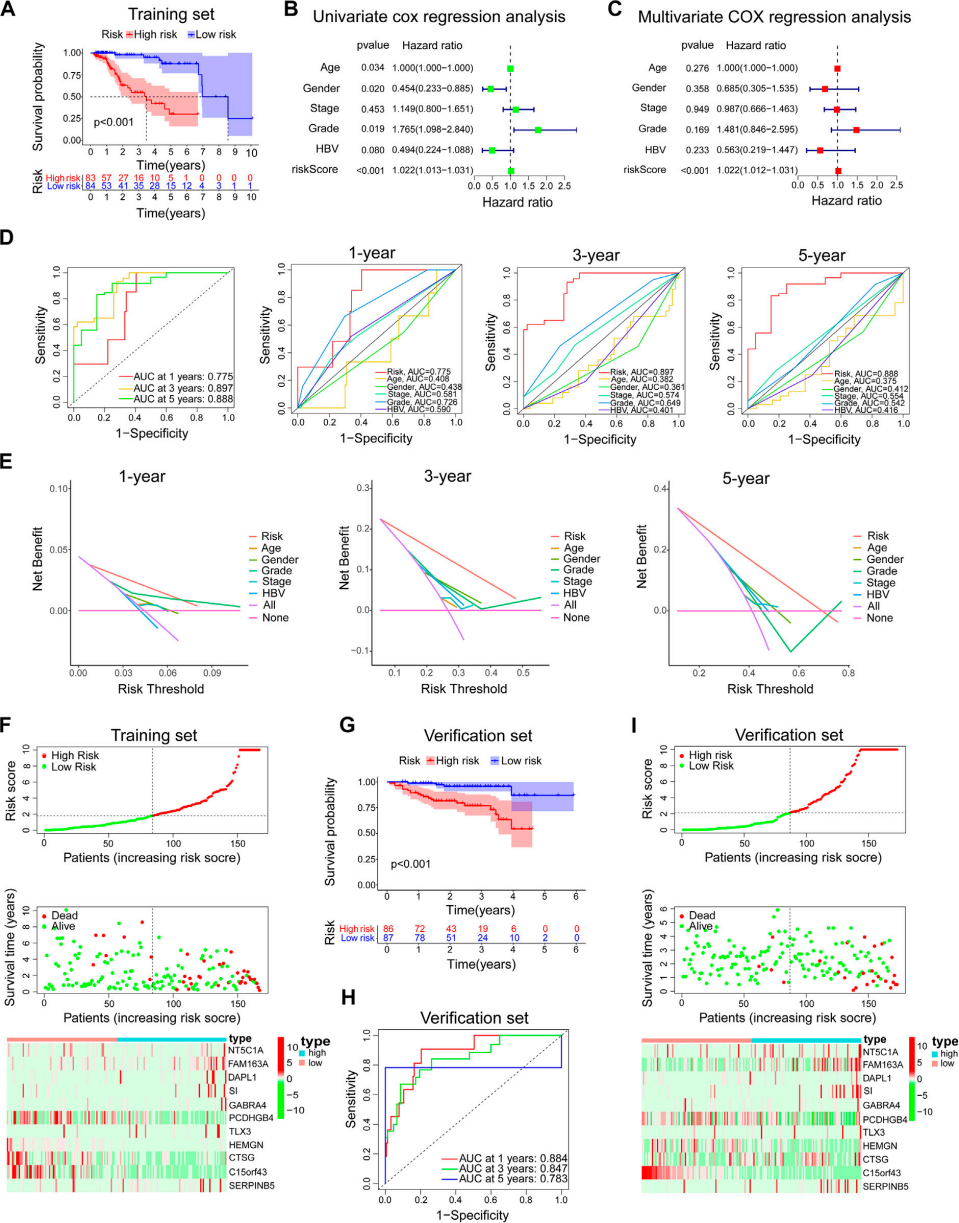
Evaluation and verification of the prediction ability of the model. In the training set, **(A)** Kaplan-Meier survival analysis between the high-risk group and low-risk group. **(B,C)** Univariate and multivariate COX regression analysis of prognostic risk model and clinical features with OS. **(D)** The time-dependent ROC curve of the prognostic risk model and the comparison with the predictive ability of major clinical features. **(E)** The DCA curve of the prognostic risk model and the comparison with the predictive ability of major clinical features. **(F)** Risk factor linkage analysis: the distribution and median of the model risk score, the distribution of dead patients in the high and low-risk group, and the expression of genes involved in the construction of the model in the high and low-risk group. In the verification set, **(G–I)** The predictive ability of the prognostic risk model was further verified by Kaplan-Meier survival analysis, ROC analysis, and risk factor linkage analysis.

**FIGURE 6:**
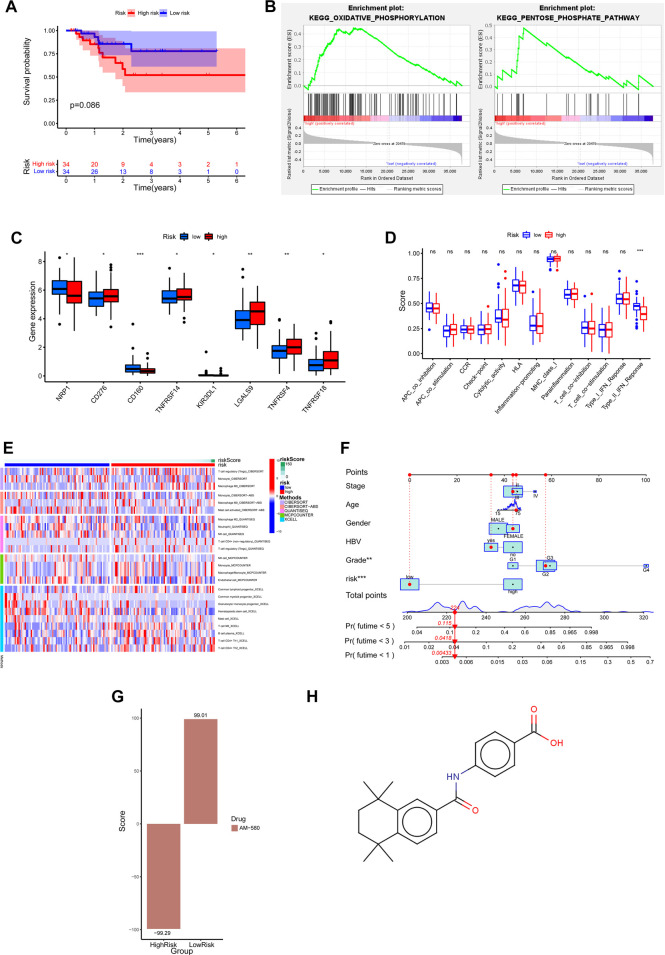
Clinical predictive application of biological differences and prognostic risk models in high and low risk groups. **(A)** Prognostic risk model of Kaplan-Meier survival curve in HCC patients with *TP53* mutant. **(B)** GSEA enrichment analysis showed that pathways were significantly associated with high risk scores. **(C)** Differential expression of common immune checkpoints between high and low risk groups. **(D)** The difference of immune-related function in high and low risk groups. **(E)** Based on five algorithms, the differences in immune cell infiltration between high and low risk groups were evaluated. **(F)** Nomograph was used to predict 1-year, 3-year and 5-year OS in patients with *TP53* wild type HCC. **(G)** The correlation between the differentially expressed genes in high and low risk groups and the expression profile of AM580. **(H)** The molecular structural formula of AM580.

In the training set, the Kaplan-Meier survival analysis showed a significant difference in prognosis between the high-risk and low-risk groups. The median survival time of the low-risk group was significantly longer than that of the high-risk group (Figure 5A). Univariate and multivariate cox regression analysis was used to evaluate independent prognostic factors in patients with *TP53* wild-type HCC. First, a univariate cox regression analysis was performed on all clinical variables and model risk scores. We found that age, sex, grade, and model risk score were closely related to the total survival time (OS) of patients with *TP53* wild-type HCC (*p* < 0.05) (Figure 5B). Through further multivariate COX regression analysis, we found that the risk score of this model could be used as an independent prognostic factor for patients with *TP53* wild-type HCC (*p* < 0.001) (Figure 5C). ROC analysis showed that the model could effectively predict the 1-year, 3-year, and 5-year survival of patients with *TP53* wild-type HCC. The predictive ability was better than other clinical features (including age, sex, grade, stage, and hepatitis B virus). A higher AUC value indicates that the model has high sensitivity and specificity (Figure 5D). At the same time, DCA analysis also showed the superiority of the model’s predictive capacity for predicting 1-year, 3-year, and 5-year survival of patients with *TP53* wild-type HCC (Figure 5E). The linkage analysis of risk factors showed that the low-risk and high-risk groups could be distinguished according to the median risk score of the model, and the death toll of the high-risk group was significantly higher than that of the low-risk group. And there are differences in the expression of genes involved in constructing the model in high and low-risk groups (Figure 5F). Next, we use *TP53* wild-type HCC samples from the ICGC database (ICGC-JP) as a verification set further to verify the predictive ability of the prognostic model. Consistent with the above analysis method, we divided the validation set into high-risk and low-risk groups according to the model risk score. Kaplan-Meier survival analysis showed that the survival probability of the high-risk group was significantly lower than that of the low-risk group (Figure 5G). ROC analysis showed that the prognostic model could also effectively predict the 1-year, 3-year, and 5-year survival of patients with *TP53* wild-type HCC (Figure 5H). The risk factor linkage analysis also draws the same conclusion as the training set (Figure 5I). As a supplement, we also evaluated the prognostic potential of the prognostic model in patients with mutant *TP53*. The risk score of each *TP53* mutant patient in the TCGA cohort was calculated using the same algorithm and grouped according to the median risk score. Kaplan-Meier survival analysis showed that the prognostic model did not maintain a good predictive performance in patients with *TP53* mutant HCC (Figure 6A). To sum up, the prognostic model is reliable in predicting the clinical prognosis of patients with *TP53* wild-type HCC.

### 3.6 Clinical predictive application of biological differences and risk scores in high and low groups

To explore which biological processes are involved in the poor prognosis of patients with *TP53* wild-type HCC in the high-risk group, we performed a GSEA analysis. The results showed that the high-risk group was highly enriched in metabolic-related biological processes, such as oxidative phosphorylation and pentose phosphate pathway (Figure 6B). This suggests that the imbalance of metabolic processes may be a factor for poor prognosis in the high-risk group of patients with wild-type HCC of *TP53*. At present, immunotherapy plays an important role in tumor therapy, and the difference in immune-related characteristics of different HCC patients may affect the effect of immunotherapy. Below, we further analyze whether there are differences in immune-related characteristics between high and low-risk groups of *TP53* wild-type HCC patients. The association analysis between joint immune checkpoints and high and low-risk groups showed that *NRP1*, *CD160*, and *KIR3DL1* were highly expressed in the low-risk group, while *CD276*, *TNFRSF14*, *LGALS9*, *TNFRSF4*, and *TNFRSF18* were highly expressed in the high-risk group (*p* < 0.05) (Figure 6C). However, the analysis of immune-related function only showed that Type_II_IFN_Reponse was enriched in the low-risk group (Figure 6D). We then used five algorithms: CIBERSORT, CIBERSORT−ABS, QUANTISEQ, MCPCOUNTER, and XCELL, to analyze the correlation between immune cell infiltration and high and low-risk groups. The results showed that T-cell regulatory (Tregs), Macrophage M0, Macrophage M2, Neutrophil, T-cell CD4^+^ (non-regulatory), Common lymphoid progenitor, T-cell NK, B-cell plasma, T-cell CD4^+^ Th1, T-cell CD4^+^ Th2 are enriched in the high-risk group, Monocyte, Mast cell activated, NK cell, Endothelial cell, Common myeloid progenitor, Granulocyte-monocyte progenitor, Hematopoietic stem cell are enriched in the low-risk group (Figure 6E). Considering the clinical utility of risk score in predicting OS in patients with *TP53* wild-type HCC, we established a Nomograph including model risk score and main clinical features to predict 1-year, 3-year, and 5-year OS rates (Figure 6F). Next, we screened the small molecular compounds with potential therapeutic significance in high-risk patients with *TP53* wild-type HCC. First, the differences in gene expression profiles between high and low-risk groups of *TP53* wild-type HCC were analyzed, and the upregulated genes of the high-risk and low-risk groups were uploaded to the CMap database for comparison. We found that the expression profile of AM580 was similar to that of upregulated genes in the low-risk group (score = 99.01) but opposite to that in the high-risk group (score = −99.29) (Figure 6G). This suggests that AM580 has potential therapeutic significance for patients with *TP53* wild-type high-risk HCC, and we provide a structural formula for AM580 (Figure 6H).

## 4 Discussion

Hepatocellular carcinoma (HCC) is the most prevalent type of primary liver cancer, and it is a fatal disease that usually occurs in the context of chronic liver disease (such as liver cirrhosis) (Craig et al., 2020). High-throughput sequencing technology has extensively promoted our understanding of hepatocellular carcinoma (HCC) biology. More knowledge of the molecular map has been transformed into new therapeutic targets and biomarkers and provides a direction for improving the prognosis of patients (Yang et al., 2021). Genomic studies have identified the prospect of HCC molecular changes; however, the most common mutations are inoperable, and only about 25% of tumors have potential targeted drivers (Llovet et al., 2018). In recent years, many studies have described the gene mutation map of HCC, which mainly includes the genome sequencing queue of clinical samples and the data mining of mutation data in public databases, such as Jean-Charles Nault et al. (2020) described the inheritance and transcription of HCC from early to advanced; Xue et al. (2019) provided a detailed genome picture of cHCC-ICC; Ding et al. (2019) described the genomic and epigenomic characteristics of primary and recurrent HCC. In this study, to more reliably describe the mutations of the main driver genes of HCC, we combined the somatic mutation data from TCGA and ICGC databases. In the Whole Exome Sequencing cohort of HCC, we described the mutational status of main driving genes in different tumor stages and the mutually exclusive coexistence between mutations. We also described the close relationship between genetic alterations and major clinical features and prognosis. It is worth noting that in our Whole Exome Sequencing cohort, most patients are male patients with hepatitis B, so it is impossible to explain whether there is an association between gender, hepatitis B virus infection, and main driver gene mutations.

Cancer is a genomic disease formed by a combination of heritable variation (usually marked by single nucleotide polymorphism (SNP)) and acquired somatic mutations. Genome-wide association studies (GWAS) and tumor sequencing studies have revealed many heritable variants and somatic drivers, which are roughly divided into oncogenes and tumor suppressor genes (Bader, 2021). A better understanding of the mutations of different genes in cancer patients is also conducive to drug development and personalized treatment. And ideally, more people with genetic predisposition will be identified before diagnosis, so that effective screening and prevention management strategies can be considered (Mendiratta et al., 2021; Rooney et al., 2023). The mutation of the tumor suppressor gene *TP53* is one of the most common mutations in HCC. After DNA damage, the cell cycle regulation and apoptosis of *TP53* mutant cells are affected, and they can escape apoptosis and transform into cancer cells when DNA damage occurs (Lai et al., 2007; Yang et al., 2020). The mutated *TP53* protein loses its tumor suppressor function simultaneously (Brosh and Rotter, 2009). *LRP1B* is one of the most changed genes in human cancer, and it is mainly considered a presumptive tumor suppressor (Príncipe et al., 2021). Its expression, mutation status, and function in cancer still need further study. It is worth noting that *TP53* mutation and *LRP1B* mutation are associated with poor prognosis in patients with HCC (Liu et al., 2012; Liu et al., 2021). In this study, compared with the wild group, both the *TP53* mutant group and *LRP1B* mutant group were significantly enriched in DNA replication, base excision repair, cell cycle, and other pathways, suggesting that the changes of these pathways may affect the immune response and other biological functions of patients with *TP53* and *LRP1B* mutations, and may also be factors leading to poor prognosis of mutant patients. In tumors, the increase of immunosuppressive cells (Treg cells and tumor-associated macrophages, *etc.*), the overexpression of immunosuppressive molecules (*CTLA-4*, *etc.*), and the decrease of tumor antigens will lead to the inability of CD8T cells to recognize cancer cells, thus promoting the occurrence and development of tumors (Zou and Chen, 2008). The analysis of immune cell infiltration in patients with *TP53*, *LRP1B* mutant, and corresponding wild-type HCC further showed the change in the proportion of infiltrating immune cells in 22 tumors. It is worth noting that except for the significant enrichment of activated NK cells in the *LRP1B* mutation group, there is no significant correlation between *LRP1B* mutation and other immune cell infiltration, which may be related to the small proportion of patients with *LRP1B* mutation in TCGA. However, relevant studies have shown that although there is no significant correlation between *LRP1B* mutation and tumor immune infiltrating cells, the expression level of *LRP1B* is associated with various immune checkpoints, immune infiltrating cells, and immune cell markers (Wang and Xiong, 2021).

The development of bioinformatics has extensively promoted the understanding of the occurrence and development of hepatocellular carcinoma, and the derivative results may promote the clinical realization of proper medical care and stratified medical care. Presently, the development of hepatocellular carcinoma gene signature is primarily based on the population. For example, Junyu Long et al. established a prognostic model for predicting OS in patients with HCC based on four mRNA (Long et al., 2018); Zhang et al. (2020) established a hepatocellular carcinoma diagnosis, prognosis and recurrence model based on three hypoxia-related genes; Liang et al. (2020) established a hepatocellular carcinoma prognostic model based on novel iron filariasis related genes, and so on. However, these studies are insufficient to meet the precise risk stratification and treatment of hepatocellular carcinoma in clinical work. Recently, Yang et al. (2021) developed a genetic feature for predicting the prognosis of hepatocellular carcinoma with *TP53* mutations. They also provided potential therapeutic targets and drugs for patients with high prognosis-associated signature scores. Zhang et al. (2021) found two directly regulated lncRNA associated with *TP53* mutations in HCC. These studies provide new insights into personalized prediction methods, but the average frequency of *TP53* mutations in HCC is about 30% (Yang et al., 2022). The prognostic model for patients with *TP53* wild-type HCC is not clear. Given this situation, we developed an adequate risk model to predict the prognosis of *TP53* wild-type HCC. As expected, the prognostic model showed good predictive ability in both the training and validation sets. The predictive power of the prognostic model was independent of other clinical factors of hepatocellular carcinoma. It is worth noting that the prognostic model does not maintain a good predictive performance in HCC patients with *TP53* mutations, which reflects the specificity of its predictive performance. Through GSEA enrichment analysis, we found that the high-risk group was highly enriched in metabolic-related biological processes, including the pentose phosphate pathway and oxidative phosphorylation. The activation of the pentose oxide phosphate pathway can increase the intracellular redox ability of cancer cells by enhancing NADPH production, thus helping tumor cells escape oxidative stress (Kowalik et al., 2017). WTp53 promotes OXPHOS and inhibits glycolysis by regulating the expression or activity of metabolic enzymes, thus enabling the tumor inhibitory activity of p53 by disrupting cancer metabolism (Kruiswijk et al., 2015). But a recent study found that WTp53 plays a carcinogenic role by promoting metabolic transformation in cancer by inhibiting OXPHOS (Kudo et al., 2020). In short, metabolism-related pathway disorders such as the pentose phosphate pathway and oxidative phosphorylation may promote the occurrence and development of hepatocellular carcinoma. In addition, we found that there were also differences in immunological characteristics in the model risk group. In the high-risk group, the infiltration of immunosuppressive cells (Treg cells and M2 macrophages, *etc.*) was higher, and the expression level of the immune checkpoint was higher. In addition, when evaluating the related immune function, we only found that the activity of type II IFN response was significantly decreased in the high-risk group, suggesting that there may be anti-tumor immune impairment in our high-risk group. These results indicate that the risk model can not only predict the prognosis of *TP53* wild-type HCC patients but also be used to predict the effect of immunotherapy. Due to the high heterogeneity of liver cancer among individuals and the lack of corresponding biomarkers, and at present, all the treatment of advanced liver cancer is population-based, it is almost impossible to find a treatment method suitable for all cases of liver cancer, and the treatment effect is not satisfactory (Yang et al., 2021). Finding tailor-made biomarkers and treatment strategies for specific populations is part of the primary purpose of this study. It is of great significance to maximize the effectiveness of treatment. This study established a quantitative map to facilitate patient counseling, decision-making, and follow-up arrangements by integrating model risk scores and significant clinical features. Finally, we found that retinoic acid nuclear receptor (RAR) agonist AM580 can be a potential therapeutic drug for high-risk patients with *TP53* wild-type HCC.

## Data Availability

The datasets presented in this study can be found in online repositories. The names of the repository/repositories and accession number(s) can be found below: https://ngdc.cncb.ac.cn/gvm/, GVM000465.
