# Supplementary material for: Prognosis and personalized medicine prediction by integrated whole exome and transcriptome sequencing of hepatocellular carcinoma
Source: Front Genet. 2023 Feb 2;14:1075347. doi: 10.3389/fgene.2023.1075347 (PMC9932713; doi:10.3389/fgene.2023.1075347)
Supplement: Supplementary file 1 [file Table1.DOCX]

Supplementary Material

# Supplementary Tables

**Supplementary Table 1.** Clinical characteristics and the main mutation genes in 125 patients with HCC.

**Supplementary Table 2.** Correlation between the main mutation genes and clinical features.

**Supplementary Table 3.** Patients with HCC in TNM I and TNM II.

**Supplementary Table 4.** Patients with HCC in TNM III and TNM IV.
